# Lipid production in association of filamentous fungi with genetically modified cyanobacterial cells

**DOI:** 10.1186/s13068-015-0364-2

**Published:** 2015-11-05

**Authors:** Ana F. Miranda, Mohamed Taha, Digby Wrede, Paul Morrison, Andrew S. Ball, Trevor Stevenson, Aidyn Mouradov

**Affiliations:** School of Applied Sciences, Royal Melbourne Institute of Technology University, Bundoora, VIC 3083 Australia

**Keywords:** Biofuel, Bioremediation, Flocculation, Fungi, Genetic modification, Renewable energy, *Synechocystis* PCC 6803, Wastewater treatment

## Abstract

**Background:**

Numerous strategies have evolved recently for the generation of genetically modified or synthetic microalgae and cyanobacteria designed for production of ethanol, biodiesel and other fuels. In spite of their obvious attractiveness there are still a number of challenges that can affect their economic viability: the high costs associated with (1) harvesting, which can account for up to 50 % of the total biofuel’s cost, (2) nutrients supply and (3) oil extraction. Fungal-assisted bio-flocculation of microalgae is gaining increasing attention due to its high efficiency, no need for added chemicals and low energy inputs. The implementation of renewable alternative carbon, nitrogen and phosphorus sources from agricultural wastes and wastewaters for growing algae and fungi makes this strategy economically attractive.

**Results:**

This work demonstrates that the filamentous fungi, *Aspergillus fumigatus* can efficiently flocculate the unicellular cyanobacteria *Synechocystis* PCC 6803 and its genetically modified derivatives that have been altered to enable secretion of free fatty acids into growth media. Secreted free fatty acids are potentially used by fungal cells as a carbon source for growth and ex-novo production of lipids. For most of genetically modified strains the total lipid yields extracted from the fungal-cyanobacterial pellets were found to be higher than additive yields of lipids and total free fatty acids produced by fungal and *Synechocystis* components when grown in mono-cultures. The synergistic effect observed in fungal-*Synechocystis* associations was also found in bioremediation rates when animal husbandry wastewater was used an alternative source of nitrogen and phosphorus.

**Conclusion:**

Fungal assisted flocculation can complement and assist in large scale biofuel production from wild-type and genetically modified *Synechocystis* PCC 6803 strains by (1) efficient harvesting of cyanobacterial cells and (2) producing of high yields of lipids accumulated in fungal-cyanobacterial pellets.

**Electronic supplementary material:**

The online version of this article (doi:10.1186/s13068-015-0364-2) contains supplementary material, which is available to authorized users.

## Background

Growing interest in the production of clean, renewable and sustainable energy has stimulated unprecedented interest in producing new generations of renewable feedstocks for biofuel product ion including plants, microorganisms and algae, tailored for composition of essential molecules that can be directly used or be converted into petrochemicals.

Microalgae have obvious advantages in the production of biodiesel and value added chemicals compared to plants, yeast and microorganisms including: (1) high growth rates (can double biomass every 8–12 h); (2) ability to produce substantial amounts of storage triacylglycerols (TAG)/lipids; (3) ability to grow in seawater, brackish water and wastewaters; (4) their application for efficient bioremediation of different types of wastewaters (animal, municipal and mining wastewaters) by removing main nutrients, C, N, P, heavy metals and microelement’s contaminations; (5) lack of competition with agricultural crops for arable lands; (6) no need for costly feedstocks such as reduced sugars (compare to heterotrophic microorganisms such as *E. coli* and yeast); (7) less labour required for their production; (8) their growth is less affected by seasonal changes in climate; and (9) their production is easy to scale [1–14].

Cyanobacteria share the same advantages as microalgae, however, unlike microalgae they do not accumulate triacylglycerols (TAGs) and their photosynthetic membranes are made of diacylglycerols (DAGs, including monogalactosyl diacylglycerols, digalactosyl diacylglycerols, phosphatidylglycerols, and sulfoquinovosyl diacylglycerols) [15, 16]. Their lipid biosynthetic metabolism is robust and does not require environmental stresses, such as starvation for redirecting of carbon flow into fatty acids production [8–10, 17–19].

In spite of the obvious advantages there exist fundamental barriers to the industrial production of biofuels from both of these microorganisms. The major challenges include: (1) high harvesting cost; (2) a sustainable and renewable nutrient supply; (3) improvement of oil content and composition; and (4) high cost of lipid extraction [4, 5, 7, 10, 11, 14, 20–22].

Fungal-assisted bio-flocculation can address most of these challenges [23–30]. It is highly efficient, and does not require added chemicals and has a low energy input requirement. Application of alternative sources of carbon from lignocellulosic wastes, nitrogen and phosphorus from wastewaters for fungal and algal growth improves the economics of biofuel production [27, 28, 31]. Secretion of a family of hydrolytic enzymes by fungal species can convert some microalgal species into the cell-wall-free protoplasts that can in turn reduce the requirements for organic solvents used for lipids extraction [27, 28].

We recently screened 33 fungal strains isolated from wastewater sludge for their lipid content and flocculation efficiency against 15 photosynthetic microalgae: photoautotrophic and heterotrophic, freshwater and marine, unicellular and multicellular, small (5 mm), large (over 300 mm), motile and non-motile [27, 28]. Some of these associations showed synergistic effects on biomass production and lipid yield. Analysis of fatty acid composition of fungal-microalgal pellets suggested that they can be tailored and optimised by co-cultivating different microalgal and fungal representatives.

Natural symbiosis between fungi and microalgae/cyanobacteria, known as lichens, have existed since plants evolved from green algae more than 400 million years [32]. More than 20 % of existing fungal species are known to be lichenized and in an obligate mutualistic association with photoautotrophic green algae, cyanobacteria, or both types of photobionts [33].

Unicellular cyanobacteria, *Synechocystis* PCC 6803, has been considered a feedstock for renewable and sustainable energy production because of its potential for large-scale biomass production due to fast growth rate, high lipid content, ability to be genetically transformed, and robustness towards a wide range of environmental conditions (including salt concentration, pH, temperature, UV light, and carbon dioxide level) [8–10, 17–19, 34–38]. Production of thylakoid membranes in *Synechocystis* is not induced by environmental or nutritional stressors and is only dependent on their biomass-production rate [19, 39].

The wild-type strain of *Synechocystis* PCC 6803, (SD100) has recently been genetically modified using two distinct strategies designed to release free fatty acids (FFAs) directly into the cultured medium (Additional file 1: Figure S1, Additional file 2: Table S1) [17, 35, 37]. The first strategy, ‘FFA Secretion’ is based on a combination of three modifications: (1) weakening of polar cell wall layers by altering surface proteins and peptidoglycan layers, thereby allowing diffusion of FFA through phospholipid layers (deletion of *sll1951* gene in SD232, SD277 and SD262); (2) preventing FFAs being channelled into competitive pathways (deletion of an *acyl*-*ACP synthesis, slr1609* gene in all SD strains) and (3) overexpression of acyl–acyl carrier protein (ACP) thioesterases (TEs) thereby directing carbon flow into the production and secretion of FFAs. Shortening of fatty acids (C8–C14) was achieved via the expression of specific plant-based TEs, from *Cinnamomum camphorum* (14∶0), *Umbellularia californica* (12∶0) and *Cuphea hookeriana* (C8∶0 and C10∶0) [35, 40]. The second approach, the ‘Green Recovery’ technology, is based on enzymatic degradation of the lipids in photosynthetic membranes that is achieved by expression of lipolytic enzymes from *Staphylococcus hyicus (shl*, SD257, SD262*)*, *Fusarium oxysporum (fol*, SD256, SD262*)* and *gpl* from guinea-pig (SD262) under control of CO_2_-limitation-inducible promoter.

We have shown for the first time that *Aspergillus fumigatus* (*A. fumigatus)* cells can efficiently flocculate the unicellular cyanobacteria *Synechocystis* PCC 6803 and its genetically modified derivatives that have been designed for secretion of FFAs to the growth media where the hydrophobic droplets of secreted FFAs were used by fungal cells as the carbon source for growth and/or for ex-novo production of lipids. The total lipid yields of the produced fungal-*Synechocystis* pellets were found to be higher than additive yields of lipids and intracellular/extracellular FFAs of the fungal and cyanobacterial components grown separately as mono-cultures. Synergistic effect of fungal-*Synechocystis* associations was also found in bioremediation rates when animal wastewater was used as an alternative source of nitrogen and phosphorus.

## Results

### *A. fumigatus* growth on different carbon sources

The cultures of filamentous fungi *A. fumigatus* produced dense spherical pellets, approximately 5–6 mm in size when grown on FGB containing 20 g/L of glucose under 150 rpm rotation (*A. fumigatus*/GLU) (Additional file 3: Figure S2). When grown on media without an extra carbon source *A. fumigatus* produced much smaller spherical pellets, approximately 1–2 mm in size (*A. fumigatus*/NEC). When grown on an alternative carbon source, 1 % treated wheat straw (TWS), *A. fumigatus* produced pellets with diameters approximately 3–4 mm (*A. fumigatus*/TWS).

*A. fumigatus/*NEC showed lowest growth rate; after 3 days growth yielding 1.3 g/L DW of biomass (Additional file 4: Figure S3). *A. fumigatus/*GLU showed the highest growth rate at 3.7 g/L DW and intermediate growth rate was recorded for *A. fumigatus/*TWS of 2.3 g/L DW. Along with differences in growth rates, *A. fumigatus* grown on different carbon sources showed differences in lipid yields. Lipid yields were correlated with generated biomasses: 0.04 mg/g DW, 0.08 mg/g DW and 0.39 mg/g DW for *A. fumigatus/*NEC, *A. fumigatus/*TWS and *A. fumigatus/*GLU, respectively.

### Flocculation of *Synechocystis* cells by *A. fumigatus*

**Fig. 1:**
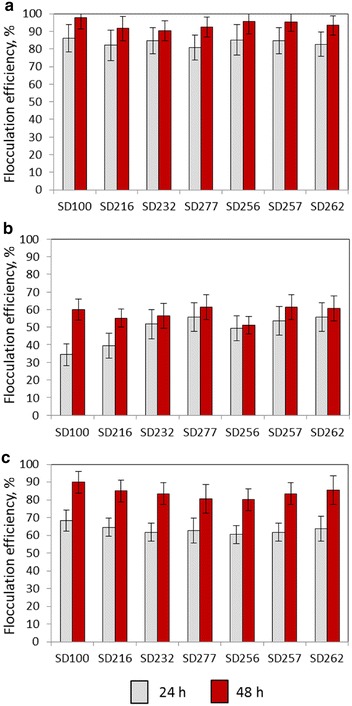
Flocculation efficiency of *Synechocystis* PCC 6803 strains by *A. fumigatus.* Flocculation of *Synechocystis* cells by *A. fumigatus/*GLU (**a**), *A. fumigatus*/NEC (**b**) and *A. fumigatus*/TWS pellets (**c**). Flocculation efficiency of *A. fumigatus* with all SD strains showed significance levels, *p* < 0.01

**Fig. 2:**
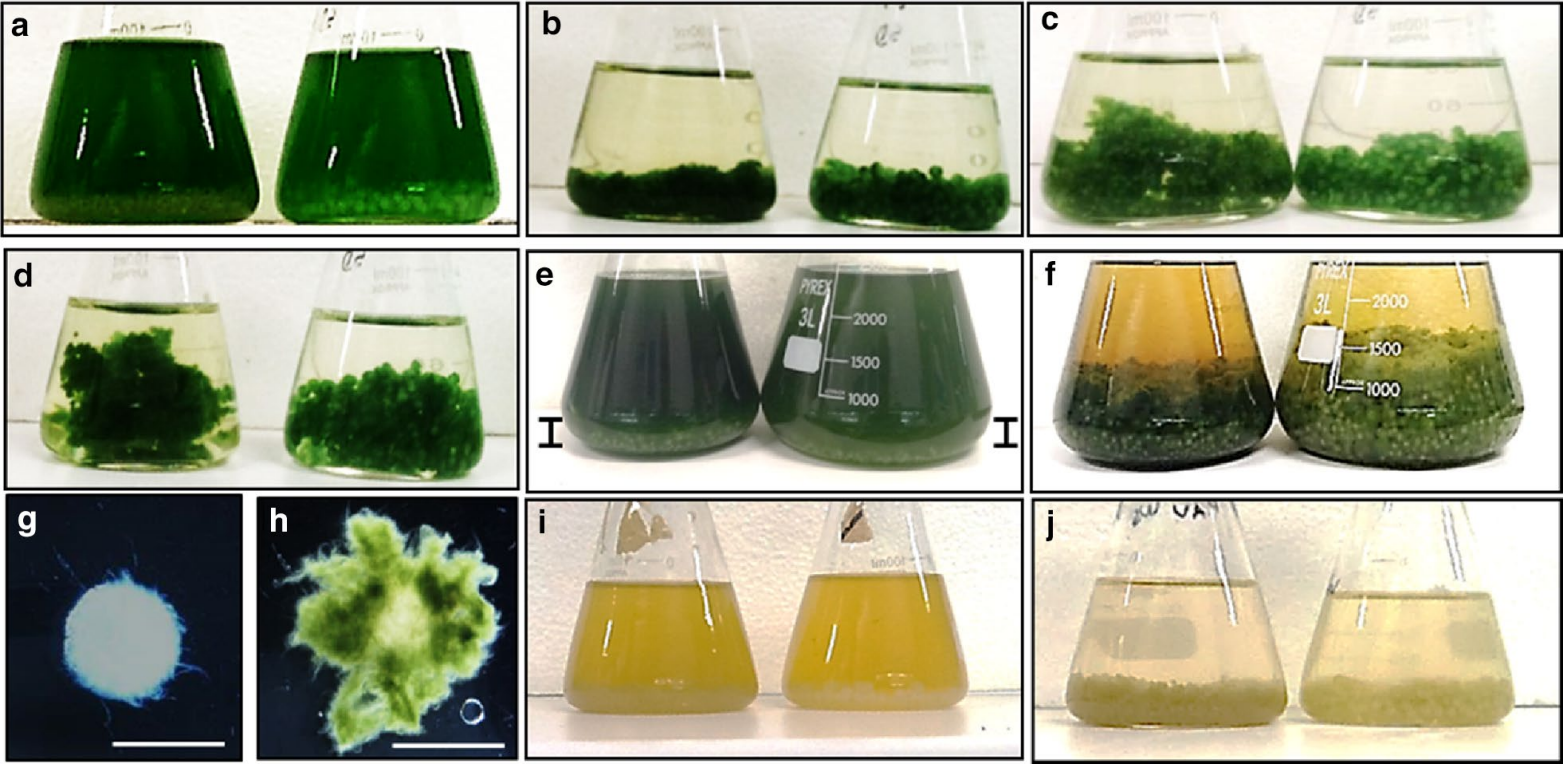
Flocculation of *Synechocystis* PCC 6803 cells by *A. fumigatus.* **a** SD100 culture mixed with *A. fumigatus/*TWS and *A. fumigatus/*GLU pellets, time = 0; Flocculation of SD100 (**b**), SD216 (**c**) and SD232 (**d**) cells with *A. fumigatus/*TWS and *A. fumigatus/*GLU pellets (*t* = 24 h); **e** Flocculation of SD277 with *A. fumigatus/*TWS and *A. fumigatus/*GLU pellets, *t* = 0; *vertical bars* show the levels of *A. fumigatus* pellets in SD277 culture; **f** same after 24 h; **g**, **h** *A. fumigatus* pellets before and after mixing with SD100 cells, respectively; **i** SD256 grown for 5 days under reduced CO_2_ conditions and mixed with *A. fumigatus/*TWS and *A. fumigatus/*GLU pellets, *t* = 0; **j** same after 24 h. In **a**–**f**, **i**–**j** *A. fumigatus/*TWS pellets were shown on *left* and *A. fumigatus*/GLU on *right*. *Scale* 5 mm

Flocculation experiments were explained in Additional file 5: Figure S4. To assess flocculation efficiency *A. fumigatus/*GLU and *A. fumigatus/*TWS pellets were mixed with wild-type and genetically modified SD cultures that had been grown to cell densities of 1.0 × 10^9^ cells/mL. Efficiency of harvesting was measured by the reduction in optical density and the numbers of uncaptured SD cells 24 h and 48 h after of co-cultivation with fungal pellets. Half maximal flocculation efficiencies (FE_50_) were calculated as the minimum amount of *A. fumigatus* cells required to harvest 50 % of the 1.0 × 10^9^ cells/mL cells. *A. fumigatus/*GLU showed up to 86 % flocculation when co-cultivated for 24 h with SD strains (Figs. 1, 2). This increased up to 97 % after 48 h co-cultivation. Mixing *A. fumigatus/*TWS with SD strains showed up to 68 % flocculation after 24 h followed by 80–90 % of flocculation after 48 h. *A. fumigatus*/NEC showed 34–56 % flocculation rates after 24 h of co-cultivation which increased to 60 % after 48 h. FE_50_ data for flocculation efficiencies is shown in Table 1.

**Table 1:** Half maximal flocculation efficiency (FE_50_) of SD strains by *A. fumigatus*

Species	*A. fumigatus*/NEC		*A. fumigatus*/GLU		*A. fumigatus*/TSW	
	FE50, gDW/l	*R* ^2^	FE50, gDW/l	*R* ^2^	FE50, gDW/l	*R* ^2^
SD100	6.01 ± 1.5	0.78	3.7 ± 0.8	0.85	5.8 ± 1.1	0.87
SD216	7.78 ± 1.6	0.81	3.9 ± 0.9	0.84	5.1 ± 1.0	0.81
SD232	7.2 ± 1.7	0.85	3.5 ± 1.1	0.78	4.0 ± 1.0	0.78
SD277	7.8 ± 1.4	0.84	3.8 ± 0.9	0.81	4.2 ± 1.1	0.85
SD256	7.78 ± 1.6	0.89	3.3 ± 0.9	0.84	5.1 ± 1.0	0.81
SD257	7.2 ± 1.7	0.85	3.5 ± 1.1	0.78	4.8 ± 1.0	0.78
SD262	6.8 ± 1.4	0.88	4.1 ± 0.9	0.81	4.2 ± 1.1	0.85

To test toxicity of products produced during cultivation of *A. fumigatus* with 1 % TWS we grew SD strains in the presence of 5 and 20 % of the media collected 72 h after incubation of *A. fumigatus* with 1 % TWS (Additional file 6: Figure S5). No obvious effects were observed in presence of 5 % TWS added to media. However, significant suppression of growth was observed in presence of 20 % TWS. To avoid this effect *A. fumigatus*/TWS pellets were washed before mixing with SD cultures. This led to no suppression effect on SD strains growth (not shown).

**Fig. 3:**
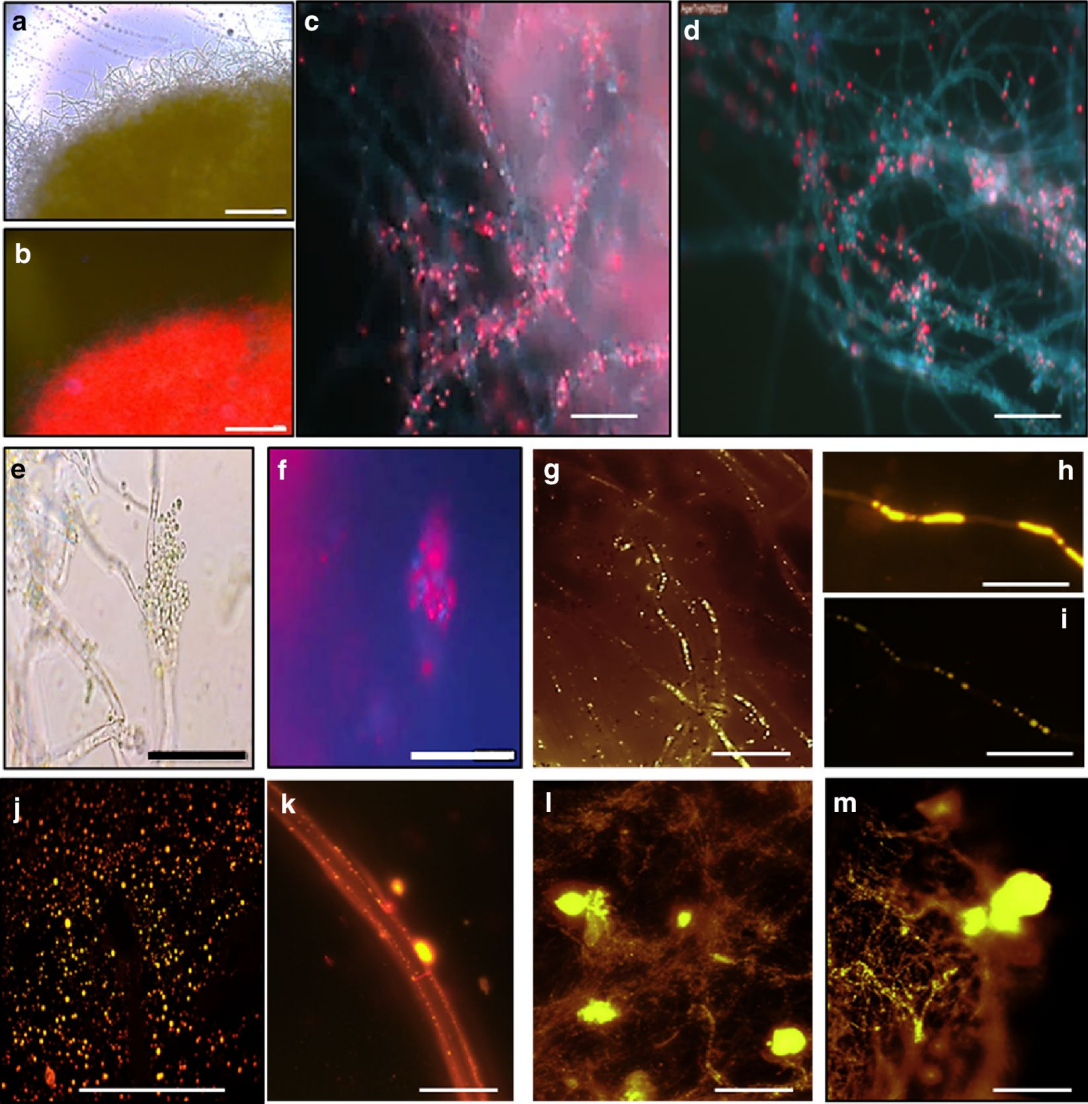
Microscopic analysis of *A. fumigatus*-*Synechocystis* associations. **a**, **b** *A. fumigatus*-SD100 pellets; **c**, **d** *A. fumigatus*-SD100 and *A. fumigatus*-SD216; **e**, **f** SD100 cells attached to *A. fumigatus* filaments; **g**, **h** *A. fumigatus/*GLU filaments stained with Nile red; **i** *A. fumigatus/*TWS filaments stained with Nile red; **j** FFA droplets secreted into growth media by SD277 growing in mono-culture; **k**–**m** FFA droplets attached to *A. fumigatus* filaments after mixing with SD232 (**k**) and SD277 (**l**, **m**) cultures at *t* = 0. *Red colour* is the autofluorescence of cyanobacterial phycobilisomes. *Scales* **a**, **b** = 1 mm; **c**–**m** = 20 µm

Detailed light and environmental scanning electron microscopy of the *A. fumigatus*-SD pellets showed that cyanobacterial cells not only trapped within fungal filaments but were clearly attached to them (Fig. 3; Additional file 7: Figure S6).

### Zeta potential and cell size measurements

The electrostatic charge distributions across the surfaces of the *Synechocystis* and *A. fumigatus* cells were evaluated by means of zeta potential values. Zeta potential values for *Synechocystis* cells grown in BG11 media showed strong negative surface charges between −22.5 mV (SD256) and −33.1 mV (SD100) (Table 2). The representatives of ‘Green recovery’ strains grown in CO_2_ enriched media showed slightly reduced negative surface charges, relative to wild type and ‘FFA secretion’ strains. *A. fumigatus* spores collected from the 3 week old plates showed strong negative zeta potential (−48 mV). However, *A. fumigatus* submerged in BG11 after growing on different carbon sources showed positive zeta potential, +2.6 mV for *A. fumigatus*/GLU; +0.9 mv for *A. fumigatus*/TWS and +1.2 mV for *A. fumigatus*/NEC. To analyse whether *A. fumigatus*-assisted flocculation of *Synechocystis* cells is effect of neutralization or reduction of their negative surface charges we mixed SD100 and SD277 with *A. fumigatus* and analysed zeta potential values of pellets after flocculation. Extensive measurements of the electrostatic charge distributions across the surfaces of the *A. fumigatus*-SD100 and *A. fumigatus*-SD277 pellets showed low negative values (from −2.5 to −5.1 mV). Measurements of cell sizes showed that all genetically modified SD cells showed slightly larger sizes (5.1–5.9 µM) than wild type SD100 cells (3.6 µM) (Table 2).

**Table 2:** Zeta potentials and sizes of microalgal, *Synechocystis* and *A. fumigatus* cells

Sample name	ZP, mV	pH	Size, nm
Algae and PCC 6803			
*C. vulgaris* ^a^	−19.0 ± 0.6	6.8	4725.3
*Isochrysis* sp.^a^	−9.0 ± 0.9	6.5	15,540
SD100^a^	−33.1 ± 2.9	7.8	3567.6
SD232^a^	−30.1 ± 2.6	6.9	5974.3
SD277^a^	−31.2 ± 1.8	7.2	5177.5
SD256^a^	−22.5 ± 2.0	7.6	5513
SD257^a^	−25 ± 1.9	7.3	5087
*A. fumigatus*			
*A. fumigatus*, spores^b^	−48.5 ± 2.6	NA	NA
*A. fumigatu*s/FGB^c^	−5.4 ± 0.6	7.3	NA
*A. fumigatu*s/GLU^d^	2.6 ± 0.6	7.3	NA
*A. fumigatu*s/TWS^d^	0.9 ± 0.5	7.7	NA
*A. fumigatu*s/NEC^d^	1.2 ± 0.9	7.8	NA
*A. fumigatus*-SD			
*A. fumigatus/*GLU-SD100^d^	−2.5 ± 0.8	7.3	NA
*A. fumigatus/*TWS-SD100^d^	−3.8 ± 1.1	7.5	NA
*A. fumigatus/*NEC-SD100^d^	−4.8 ± 1.9	7.7	NA
*A. fumigatus/*GLU-SD277^d^	−3.2 ± 1.1	7.3	NA
*A. fumigatus/*TWS-SD277^d^	−4.4 ± 1.5	7.7	NA
*A. fumigatus/*NEC-SD277^d^	−5.1 ± 1.9	7.8	NA

^a^Zeta potential of cells grown in their growth media

^b^Zeta potential of *A. fumigatus* spores collected from 3 week old plates

^c^Zeta potential of cells grown in FGB media

^d^Zeta potential of cells grown in BG11

### Biomass, lipid and FFA production in mono-cultured *A. fumigatus* and *Synechocystis* strains

Before mixing with SD cultures mono-cultured *A. fumigatus*/GLU pellets had a lipid content of 11.1 % (*t* = 0, Table 3). No secreted FFAs were detected in growth media. After an additional 24 h growth (control, *t* = 24 h) the biomass of *A. fumigatus* pellets was slightly increased (from 1.0 to 1.77 gDW/L). Not surprisingly, *A. fumigatus*/TWS pellets showed a lower lipid content, 3.4 % DW (*t* = 0, Table 3). After a further 24 h of growth (control, *t* = 24) biomass of *A. fumigatus* pellets increased to 1.5 gDW/L (4.2 % DW).

**Table 3:** Biomass and lipids concentrations in *A. fumigatus* and ‘FFA Secretion’ strains grown in mono-cultures and after co-cultivations

Species	Biomass (g/L)	Lipid/intracellular FFA yield (mg/g DW)	Lipid/intracellular FFA concentration (% DW)	Extracellular FFA, yield (mg/L)	Extracellular FFA concentration (% DW)	Biomass (g/L)	Lipid/intracellular FFA yield (mg/g DW)	Lipid/intracellular FFA concentration (% DW)	Extracellular FFA, yield (mg/L)	Extracellular FFA concentration (% DW)
*A. fumigatus*	*t* = 0 h					Control, *t* = 24 h				
*A. fumigatus*/GLU	1.04 ± 0.14	115.64 ± 16.52	11.11 ± 1.57	ND	NA	1.77 ± 0.25	188.26 ± 26.89	10.63 ± 1.51	ND	NA
*A. fumigatus*/TWS	1.20 ± 0.17	41.49 ± 5.92	3.43 ± 0.49	ND	NA	1.5 ± 0.21	64.22 ± 9.17	4.28 ± 0.61	ND	NA
SD species	*t* = 0 h					Control, *t* = 24 h				
SD100	1.4 ± 0.2	1.8 ± 0.25	0.12 ± 0.01	0.41 ± 0.05	0.029 ± 0.01	1.7 ± 0.24	1.85 ± 0.26	0.10 ± 0.01	0.89 ± 0.12	0.02 ± 0.01
SD216	1.2 ± 0.17	28.92 ± 4.13	2.41 ± 0.34	60.8 ± 8.68	5.06 ± 0.72	1.3 ± 0.18	34.6 ± 4.94	2.66 ± 0.38	61.9 ± 8.84	4.76 ± 0.68
SD232	1.1 ± 0.15	21.78 ± 3.11	1.98 ± 0.28	94.3 ± 13.47	8.57 ± 1.22	1.4 ± 0.2	28.42 ± 4.06	2.03 ± 0.29	81.3 ± 11.61	5.80 ± 0.82
SD277	1.1 ± 0.18	9.71 ± 1.38	0.88 ± 0.12	127.1 ± 18.15	11.55 ± 1.65	1.3 ± 0.18	12.71 ± 1.81	0.97 ± 0.13	135.1 ± 19.3	12.23 ± 1.74
*A. fumigatus*-SD	*A. fumigatus/*GLU-SD pellets, 24 h					*A. fumigatus/*TWS-SD pellets, 24 h				
SD100	4.5 ± 0.64	250.1 ± 35.72	5.55 ± 0.79	ND	NA	2.8 ± 0.4	59.1 ± 8.44	2.11 ± 0.30	ND	NA
SD216	5.1 ± 0.72	389.43 ± 55.63	7.63 ± 1.09	1.8 ± 0.25	0.05 ± 0.01	3.99 ± 0.57	192.22 ± 27.46	4.81 ± 0.68	ND	NA
SD232	6.22 ± 0.88	499.73 ± 71.39	8.02 ± 1.14	3.3 ± 0.47	0.07 ± 0.01	4.5 ± 0.64	211.05 ± 30.15	4.69 ± 0.67	ND	NA
SD277	7.6 ± 1.085	635.18 ± 90.74	8.35 ± 1.19	5.3 ± 0.75	0.09 ± 0.013	5.2 ± 0.74	299.8 ± 42.82	5.76 ± 0.82	ND	NA

Prior to mixing with *A. fumigatus* pellets the *Synechocystis* PCC 6803 strains showed a wide range of lipid, intracellular and secreted FFA concentrations (*t* = 0, Table 3). SD100 strain showed lowest concentrations of lipids/intracellular FFAs accounted up to 0.1 % of DW along with very low concentration of secreted FFAs, 0.41 mg/L (0.03 % DW). After additional 24 h (control, *t* = 24 h, Table 3) its biomass was increased up to 1.7 g/L producing to 0.9 mg/L (0.03 % DW) of secreted extracellular FFAs.

Constitutive expression of a bacterial TE gene (*tesA*) in *Synechocystis* strain SD216 led to increased levels of lipids, intracellular FFAs and secreted FFAs. At *t* = 0 h this strain accumulated lipids/internal FFAs at concentration of 2.4 % DW, at a yield of 28.9 mg/g (Table 3). Concentration of secreted FFAs was 5.1 % DW. After an additional 24 h biomass was increased up to 1.3 gDW/L producing 34.6 mg/g of lipids/intracellular FFAs (2.6 %). Concentration of secreted FFAs was 61.9 mg/L representing 4.8 % of DW (control, *t* = 24 h).

Weakening of the cell walls in SD232 associated with the expression of two additional TEs, *ChFatB2* and *UcFatB1* led to a 33 % increase in the concentrations of secreted FFAs compare to SD216 strain producing 94.3 mg/L of FFAs (8.6 %). However, concentration of lipids/intracellular FFAs was reduced up to 1.9 % producing a yield of 21.8 mg/g. The SD277 strain expressing codon-optimized *tesA* gene along with constitutive expression of three plant TEs; *ChFatB2*, *CCFatB1* and *UcFatB1*, showed more than a 1.3-fold increase in secretion of FFA (12 % DW) compare to SD232. This was correlated with 2.2-fold decrease in intracellular lipid concentration (0.88 %). Growth for an additional 24 h resulted in an increase in biomass and in yield of lipids and both internal FFAs and secreted FFAs. This was observed in both SD232 and SD277 strains (control, *t* = 24 h, Table 3).

For ‘Green Recovery’ experiments bubbling with 4 % CO_2_ was stopped 3 days before mixing with fungal pellets (see “Methods” for details). At this stage strains SD256 and SD257 accumulated 39 mg/g (4.3 %) and 42 mg/g (5.1 %) of lipids/intracellular FFAs, respectively. Amount of secreted FFAs from these cells was very low, 1.7 mg/L (0.2 %) and 0.9 mg/L (0.1 %), respectively (4 % CO_2_, Table 4A). After 3 days growth under CO_2_ depleted conditions cultures started to decolorize, changing from blue/green to yellow as a result of degradation of photosynthetic membranes. At this time, (CO_2_ limitation, *t* = 0 h, Table 4B), the total biomass of cells (cell debris) was reduced, and in strains SD256 and SD257 concentrations of lipids/intracellular FFAs were just 2.7 and 2.9 %, respectively. This drop was associated with a 9.9-fold and a 21-fold increase in the concentrations of secreted FFAs from SD256 and SD257, respectively. The membrane damage, as a result of the expression of lipase genes in these strains led to FFA recoveries of 29 and 32 % from cells of SD256 and SD257, respectively. Growing SD strains for a further 24 h did not change the lipid and FFA secretion profiles (CO_2_ limitation, *t* = 24 h, Table 4C).

**Table 4:** Biomass and lipids concentrations in *A. fumigatus* and ‘Green Recovery’ strains grown in mono-cultures and co-cultures

Strains	Biomass (g/L)	Lipid/intracellular FFA yield (mg/g DW)	Lipid/intracellular FFA concentration (% DW)	Extracellular FFA, yield (mg/L)	Extracellular FFA concentration (% DW)	FFA recovery from lipids, %
SD strains						
(A) 4 % CO_2_						
SD256	0.89 ± 0.12	39 ± 5.57	4.38 ± 0.62	1.7 ± 0.6	0.19 ± 0.1	NA
SD257	0.82 ± 0.10	42 ± 6.1	5.12 ± 09	0.9 ± 0.1	0.11 ± 0.2	NA
SD262	0.87 ± 0.12	52 ± 6.8	5.97 ± 0.89	26.9 ± 4.1	3.09 ± 0.44	NA
(B) CO_2_ limitation (*t* = 0)						
SD256	0.65 ± 0.15	17.8 ± 5.71	2.73 ± 0.51	12.3 ± 2.6	1.89 ± 0.1	29.28 ± 8.1
SD257	0.71 ± 0.17	20.32 ± 8.1	2.86 ± 0.91	16.7 ± 3.8	2.35 ± 0.4	32.11 ± 9.7
SD262	1.0 ± 0.2	36.11 ± 8.1	3.61 ± 0.71	48.3 ± 11.9	4.83 ± 1.3	NA
(C) CO_2_ limitation (control, *t* = 24)						
SD256	0.60 ± 0.11	16.3 ± 4.34	2.61 ± 0.74	13.4 ± 3.3	2.24 ± 0.3	34.35 ± 8.3
SD257	0.70 ± 0.15	18.47 ± 8.1	2.86 ± 0.91	18.0 ± 4.9	2.57 ± 1.5	33.85 ± 12.1
SD262	1.1 ± 0.6	30.46 ± 11.1	2.7 ± 0.47	52.3 ± 12.9	4.75 ± 1.2	NA
*A. fumigatus/*GLU-SD						
(D) Co-cultivation of *A. fumigatus/*GLU with SD strains (*t* = 24 h)						
SD256	3.9 ± 0.6	280 ± 41.1	7.17 ± 1.1	ND	NA	NA
SD257	4.1 ± 0.5	302 ± 38.9	7.36 ± 1.1	ND	NA	NA
SD262	5.8 ± 1.7	468.35 ± 68.8	8.08 ± 1.2	ND	NA	NA
*A. fumigatus*/TWS-SD						
(E) Co-cultivation of *A. fumigatus/*TWS with SD strains (*t* = 24 h)						
SD256	3.29 ± 0.4	112.22 ± 16.1	3.81 ± 0.5	ND	NA	NA
SD257	3.5 ± 0.6	140.05 ± 25.1	4.00 ± 0.7	ND	NA	NA
SD262	4.6 ± 0.5	239.8 ± 53.9	5.21 ± 0.4	ND	NA	NA

SD262 containing gene families involved in both technologies showed secretion of 26.9 mg/l of FFAs (3.1 % DW) before CO_2_ limitation as a results of ‘FFA Secretion’ machinery (Table 4A). Up-regulation of three lipase genes after 3 days of CO_2_ limitation has contributed to secretion of 48.3 mg/l (4.8 % DW) of FFAs (Table 4B). Additional 24 h of growth did not affect FFA’s secretion rates (CO_2_ limitation, *t* = 24 h, Table 4C).

### Biomass, lipid and FFA production after co-cultivation of *A. fumigatus/*GLU with *Synechocystis* strains

After 24 h of co-cultivation of *A. fumigatus*/GLU with SD100 the biomass of *A. fumigatus*/GLU-SD100 pellets was 1.4-fold higher than additive biomasses of both components grown in monocultures for 24 h (Table 3; Additional file 5: Figure S4, Additional file 8: Figure S7). This was correlated with increased production of lipids in these pellets: 1.3-fold higher than additive lipid, intracellular FFAs plus extracellular FFAs in both fungal and *Synechocystis* SD100 components grown in monocultures.

With consideration of the observed 81–86 % efficiency of flocculation of *Synechocystis* cells the total biomasses *A. fumigatus*/GLU-SD216, *A. fumigatusGLU*-SD232 and *A. fumigatus/GLU*-SD277 pellets were increased 1.8-, 2.1- and 2.7-fold, respectively compare to the total (additive) biomass of pairs of components grown in monocultures (Table 3; Additional file 8: Figure S7). This was correlated with increases in yield of lipid/intracellular FFAs which were accounted up to 1.4-, 1.7- and 1.9-fold higher than the additive yields of lipid/intracellular FFAs and extracellular FFAs of mono-cultured *A. fumigatus* and SD216, SD232 and SD277 strains, respectively. FFAs droplets which were clearly seen in growth media and were attached to the fungal filaments immediately after mixing (Fig. 3) were not detected after 24 h of co-cultivation (data not shown).

Flocculation of strains SD256 and SD257 strains grown under CO_2_ limited conditions with *A. fumigatus* is shown in Fig. 2 and Additional file 9: Figure S8. Again, with consideration of the observed 83–85 % efficiency of flocculation total biomasses *A. fumigatus*/GLU-SD256, *A. fumigatus*-SD257 and *A. fumigatus*-SD262 pellets were increased 1.6-, 1.7- and 12-fold, respectively, over monoculture additive biomasses (Table 4D; Additional file 8: Figure S7). This was correlated with 1.1-, 1.2- and 1.5-fold increases in yields of lipid/intracellular FFAs in SD256, SD257, SD262 pellets, respectively, compare to the additive yields of lipid/intracellular FFA and extracellular FFA extracted from mono-cultured *A. fumigatus* and the *Synechocystis* strains.

### Biomass, lipid and FFAs production after co-cultivation of *A. fumigatus/*TWS with *Synechocystis* strains

Total biomasses and extracted lipids/internal FFAs from *A. fumigatus*/TWS-co-cultivated with *Synechocystis* strains SD216, SD232 and SD277 pellets were all also higher than the additive biomasses and yields of lipids/intracellular FFAs of the fungal and cyanobacterial components grown in monocultures. With consideration of 61–68 % efficiency of flocculation after 24 h total biomasses *A. fumigatus*/TWS- SD216, *A. fumigatus*/TWS-SD232 and *A. fumigatus*/TWS-SD277 pellets were increased 1.5-, 1.6- and 2.1-fold, respectively. This correlated with 1.1-, 1.1- and 1.3-fold increases in the yields of extracted lipids/internal FFAs compare to additive yields of lipids/internal FFAs and secreted FFAs of the components grown 24 h as monocultures (Table 3; Additional file 8: Figure S7).

For ‘Green Recovery’ strains total biomasses and yields of extracted lipids/internal FFA from *A. fumigatus*/TWS-SD256, *A. fumigatus*/TWS-SD257 and *A. fumigatus*/TWS-SD262 pellets were also all higher than additive biomasses and lipids/FFAs yields of the fungal and cyanobacterial components grown in mono-cultures (Table 4E; Additional file 8: Figure S7). Again, taking into consideration the observed 61–68 % efficiency of flocculation after first 24 h total biomasses of pellets were increased 1.4-, 1.6- and 1.9-fold, respectively. This was correlated with respective 1.0-, 1.2- and 1.4-fold increase in the yields of extracted lipids/internal FFA compare to additive yields of lipids/internal FFA and secreted FFA of the components grown 24 h in monoculture.

### Fatty acids composition in *A. fumigatus*-SD pellets

**Fig. 4:**
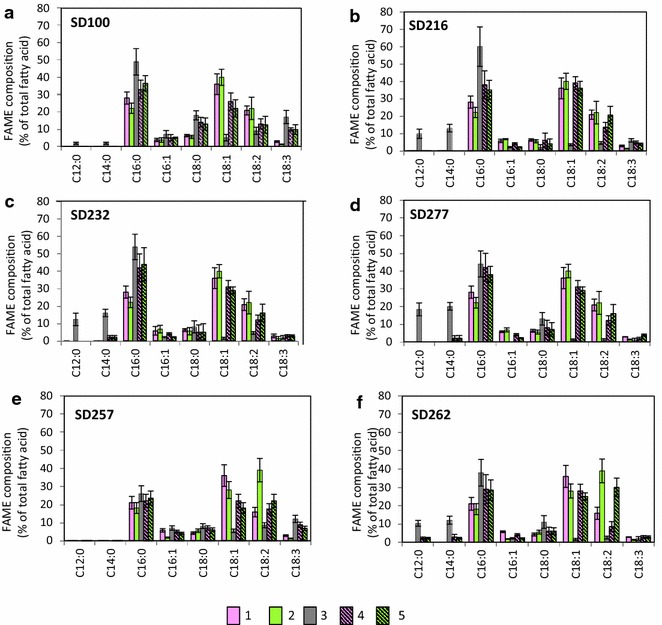
Fatty acids composition of lipids in *A. fumigatus*, SD strains and *A. fumigatus*-SD pellets. *1* *A. fumigatus*/TWS; *2* *A. fumigatus*/GLU; *3* SD strains; *4* *A. fumigatus*/TWS-SD pellets; *5* *A. fumigatus*/GLU-SD pellets

Fatty acid composition (measured as fatty acids methyl esters, FAMEs) of *A. fumigatus*/GLU and *A. fumigatus*/TWS pellets, *Synechocystis* species and pellets of *fumigatus*/TWS-*Synechocystis* strains are shown in Fig. 4. Fatty acid composition of *A. fumigatus*/GLU was dominated by oleate, C18:1 (ca 30 %) linoleate, C18:2 (ca 30 %), and palmitate, C16:0 (ca 20 %) [23, 27, 28]. *A. fumigatus*/TWS pellets showed similar proportions of these fatty acids.

Intracellular lipid and FFA’s composition of *Synechocystis* SD100 strain was also dominated by palmitate, C16:0 (49 %) [24]. Unlike *A. fumigatus* cells, the *Synechocystis* SD100 showed high proportions of stearate, C18:0 (18 %) and linolenate, 18:3 (10 %). Very low concentrations of short fatty acids were observed in the SD100 strains, with only 2 % of both, lauric acid (C12:0) and myristic acid (C14:0).

The ‘FFA Secretion’ strains, SD216, SD232 and SD277 all showed increased levels of short fatty acids, C12:0 and C14:0 relative to the SD100 strain with the concentration of C12:0 increased 5.4-, 6.8- and 10-fold, respectively. The levels of C14:0 were also increased 7.2-, 8.8- and 11-fold, respectively. This increase was associated with decreases in levels of longer chain fatty acids, C18:0, C18:1, C18:2 and C18:3. The ‘Green Recovery’ strains, SD256 and SD257 had fatty acid compositions that resembled wild type SD100 showing elevated concentrations of unsaturated fatty acids, C18:1, C18:2 and C18:3. SD262 showed fatty acid composition of both SD100 and SD232 strains accumulating both unsaturated and short-chain fatty acids.

Fatty acid compositions of the *A. fumigatus*-*Synechocystis* pellets are also shown in Fig. 4. In all cases, both *A. fumigatus* and SD strains co-contributed to the level of palmitate (C16:0). *A. fumigatus* was a main contributor of the oleate, (C18:1) and linoleate (C18:2). *Synechocystis* SD100 and SD257 were the main contributors of the linolenate (C18:3).

**Fig. 5:**
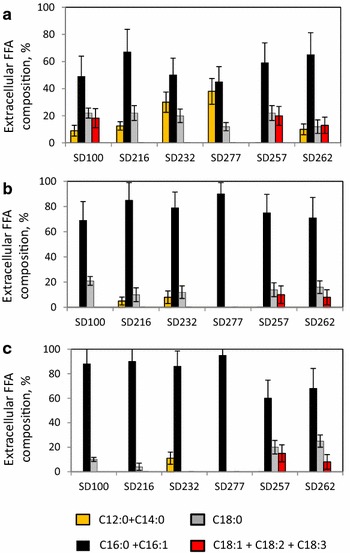
Composition of FFA secreted from *A. fumigatus*, SD strains and *A. fumigatus*-SD pellets. **a** SD strains; **b** *A. fumigatus*/TWS-SD pellets; **c** *A. fumigatus*/GLU-SD pellets

Composition of extracellular FFAs secreted from genetically modified *Synechocystis* SD strains resembled their intracellular lipid and FFAs composition, showing high levels of C16:0 and C18:0, along with short-chain fatty acids (Fig. 5). Composition of FFAs that accumulated in media containing *A. fumigatus*-*SD*216, *A. fumigatus* -SD232 and *A. fumigatus*-*SD*277 pellets was mainly C18:0 and was enriched in short fatty acids, C:12 and C14:0 with practically no detectable longer FFAs (longer than C:18). Composition of FFAs secreted from *Synechocystis* SD257 and SD262 strains was similar to composition of FFA secreted from the *Synechocystis* SD100 and SD232 strains.

### Swine wastewater as an alternative source of nutrients for *A. fumigatus*-SD100 pellets

**Fig. 6:**
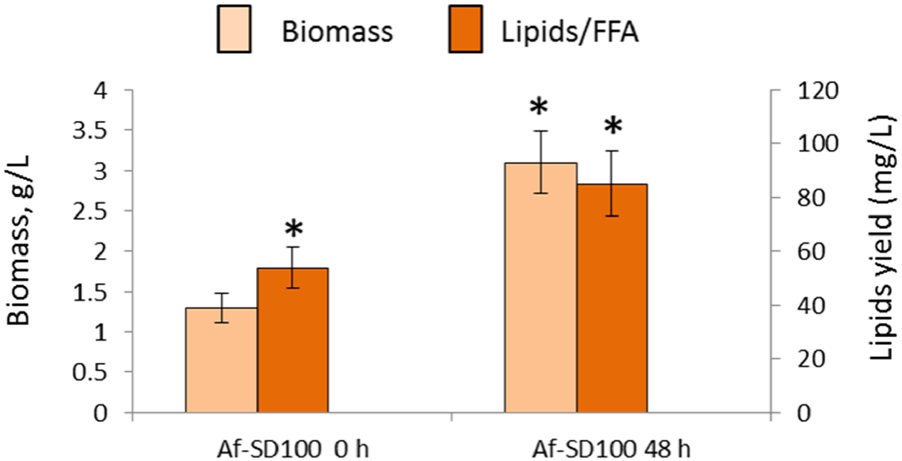
Biomass and lipid production in *A. fumigatus*-SD100 pellets grown in 25 % swine wastewater. Af-SD100: *A. fumigatus*-SD100 pellets. Significance levels: *p < 0.05

We assessed the ability of *A. fumigatus*-SD100 pellets for growth and absorption the nitrogen and phosphorus (NH_4_^+^ and PO_4_^−3^) from swine wastewater (SWW) (Table 5; Additional file 10: Figure S9). For these experiments, the swine wastewater was diluted to either 10 or 25 % with tap water. After 48 h of growth of *A. fumigatus*-SD100 pellets in 25 % wastewater the concentration of NH_4_^+^-N reduced from 164.3 to 18.2 mg/L (89 %) and the concentration of PO_4_^−3^-P reduced from 38.7 to 9.8 mg/L (75 %). This removal efficiency was higher than achieved separately by *Synechocystis* SD100 (30 % for NH_4_^+^-N and 26 % for PO_4_^−3^-P) and by *A. fumigatus* (52 and 45 %, for NH_4_^+^-N and PO_4_^−3^-P respectively). In 10 % SWW both nutrients were almost completely removed after 48 h incubation with *A. fumigatus*-SD100 alone (98 % removal for NH_4_^+^-N and 84 % removal of PO_4_^−3^-P). Nutrient uptake by *A. fumigatus*-SD100 pellets led to 2.3-fold increase in their biomass production after 48 h of treatment and this correlated with a 1.5-fold increase in lipid yield (Fig. 6).

**Table 5:** Concentrations of nutrients in 25 % swine wastewater before and after treatment with *A. fumigatus,* SD100 and *A. fumigatus*-SD100 pellets

ASW			*A. fumigatus*		SD100		*A. fumigatus*-SD100	
Concentrations	NH4-N, mg/L	PO4-P, mg/L	NH4-N, mg/L	PO4-P, mg/L	NH4-N, mg/L	PO4-P, mg/L	NH4-N, mg/L	PO4-P, mg/L
ASW, 100 %	680.7 ± 23.1	145.4 ± 13.7	NA	NA	NA	NA	NA	NA
ASW, 25 %	164.3 ± 13.2	38.7 ± 3.4	78.8 ± 8.2	21.2 ± 3.6	114.2 ± 12.1	28.5 ± 5.3	18.2 ± 6.1	9.8 ± 3.0
ASW 10 %	66.1 ± 4.3	16.1 ± 3.0	25.2 ± 2.8	8.0 ± 2.9	44.3 ± 4.2	11.9 ± 2.2	1.1 ± 0.9	2.5 ± 0.8

## Discussion

### Biomass and lipid production in *A. fumigatus*-*Synechocystis* associations

In this study we used the model cyanobacterium, *Synechocystis* sp. PCC6803 and derivative strains that have been genetically modified either for secretion of FFAs or for recovery of FFAs from photosynthetic lipids [17, 35]. Accumulation of FFA droplets in growth media by these strains was up to 12 % DW for the ‘FFA Secretion’ and 3.6 % DW for the ‘Green Recovery’ genetically modified strains. Whilst the “Green Recovery’ strains displayed lower levels of secreted FFAs, these were however up to 103-fold higher than FFAs secretion levels for the wild-type, *Synechocystis* sp. PCC6803 SD100 strain.

In spite of the obvious attractiveness of both metabolic reprogramming strategies the extraction with organic solvents of secreted FFAs from large amounts of water with organic solvents will be challenging and may not be economically viable. Apart from secreted FFAs, substantial biomass of cyanobacterial cells (or their debris) produced in both systems also contributes to total yields of bio-oil production [17, 35]. However, harvesting of cyanobacterias cells for a cost-effective large scale biofuel production will face the same challenges as those faced by the microalgal industry.

Our recent study showed that *A. fumigatus* can efficiently flocculate a large number of microalgal species, including seawater and freshwater species [27, 28]. Representatives of other *Aspergillus* species also showed high flocculation rates with freshwater microalgae *C. vulgaris* [23–26, 29, 41]. Fungal-assisted flocculation of marine microalgae, *Nannochloropsis* sp was described by [30].

In this research it was shown that *A. fumigatus* can harvest up to 80–86 % of *Synechocystis* cells after the first 24 h co-cultivation and almost 100 % after 48 h with FE_50_ around 3.6 gDW/L. Interestingly, in the fungal-SD pellets the *Synechocystis* cells were shown not only to be entrapped within the scaffolds of fungal filaments but clearly attached to them (Fig. 3). Similar types of fungal-algal interactions were described in our previous papers [27, 28]. Although the detailed mechanism of this type of fungal-algal interaction is unclear, the interaction between oppositely charged surfaces may enable microalgal attachment to the fungal cell wall. The microalgae possess a negative surface charge due to the presence of proton-active carboxylic, phosphoric, phosphodiester, hydroxyl and amine functional groups. The zeta potential of microalgae was found to be within the range from −10 to −35 mV [42]. Using coagulating agents it was shown that reduction of the magnitude of the zeta potential to approximately −10 mV and below is required for removal of four algae species *A. formosa, Melosira sp., M. aeruginosa*, and *C. vulgaris* [42].

We found that the electrostatic charge distribution across the surface of the cells of *A. fumigatus* cells depend on the age of the conidia/spores and pH. Spores collected from the agar plates showed strong negatice surface charge (−48.5 mV). Being submerged in BG11 surface charges of *A. fumigatus* were positive (+0.9 to +2.6 mV). Similar results were shown for *B. bassiana* where zeta potential for aerial conidia varied from +22 to −30 mV when pH values were ranging from 3 to 9 [43]. For submerged conidia the net surface charge was ranged from +10 to −13 mV. And much less variation observed for spores, +4 to −4 mV. The charge difference between *Aspergillus flavus* (+46.1 mV) and microalgae cells (−23.7 mV) was suggested to be essential for their flocculating interaction [41]. In our experiments *Synechocystis* cells showed strong negative surface charges.

Differences in surface charges between SD cells and fungal cells in BG11 supplemented with different carbon sources did not always correlated with their flocculation rates and FE50 values. *A. fumigatus*/GLU cells showed more positive zeta potential (+2.6 mV) than *A. fumigatus*/TWS (+0.9 mv) which correlates with differences in their flocculation rates and FE50 values. However, *A. fumigatus*/NEC showed more positive zeta potential (+1.2 mV) that *A. fumigatus*/TWS which does not correlates well with its lower flocculation efficiency and higher FE50 value. However, neutralization rates as a result of interactions between strongly negative surface potentials of SD cells and positive surface charges *A. fumigatus* cells growing on different carbon sources correlated with their flocculation efficiencies and FE50 values. Zeta potential of the *A. fumigatus*/GLU-SD100 pellets is less negative, (−2.5 mV) than the zeta potential of the *A. fumigatus*/NEC-SD100 (−4.8 mV). Zeta potential of the *A. fumigatus*/TWS-SD100 showed intermediate value (−3.8 mv). Similar results were obtained for the A. fumigatus-SD277 pellets. It is not clear whether this charge difference is enough to keep *Synechocystis* cells strongly attached to filaments. Our previous research showed that *A. fumigatus* can also efficiently trap highly motile microalgal strains such as *C. reinhardtii* and *T. chuii* [27, 28]. This is difficult to explain only by differences in their surface charges. Moreover, observation that microalgal and *Synechocystis* cells not only attach to *A. fumigatus* fungal filaments, but also to each other suggests that produced cell wall-free protoplasts have lost their negative charges. Production of protoplasts was previously shown to be triggered by secretion of cellulases by fungal cells [27, 28]. Fungal cells can secrete a cocktail of concentrated exopolysaccharide molecules during interaction with other microrganisms [28, 44, 45]. This suggests that as an alternative or an additional scenario *Synechocystis* cells can be entrapped by cocktails of ‘sticky’ exopolysaccharides secreted by *A. fumigatus.* Metabolomic analysis of media after co-cultivation of *A. fumigatus* with *Synechocystis* with microalgal strains could reveal more information about the biochemistry of fungal-assisted flocculation.

The lipid and FFA yields and their respective compositions in fungal-*Synechocystis* sp. PCC6803 pellets showed complex profiles. This complexity may be a reflection of at least two factors: (1) lipid and FFA concentrations in fungal and *Synechocystis* cells prior to and during co-cultivation and (2) the efficiencies of *Synechocystis* cell flocculation by *A. fumigatus* [23–27, 29, 46].

Fatty acid composition of all fungal-SD pellets obviously reflected the compositions of both fungal and *Synechocystis* components. Similar results were described for a number of fungal-assisted microalgal flocculation [23–29]. Being a major contributor of lipids in *A. fumigatus*-SD pellets, fungal cells were also the main contributor of medium-length fatty acids, oleates, C18:1 and linoleates, C18:2. In all instances, both *A. fumigatus* and *Synechocystis* strains contributed to the level of palmitates (C16:0), and SD232, SD277 and SD262 strains were solo contributors of short-length fatty acids, C12:0 and C14:0. The composition of saturated, extracellular FFAs secreted from genetically modified *Synechocystis* strains resembled their compositions in lipids and intracellular FFAs: they were enriched by C18:0 together with low levels of short fatty acids. Interestingly, unsaturated fatty acids palmitoleates (C16:1), oleates, (C18:1), linoleates, (C18:2) and linolenates, (18:3) were missing in *A. fumigatus* pellets in ‘FFA secretion” strains.

### De novo lipid production in *A. fumigatus*-*Synechocystis* associations

Most microorganisms including fungi have robust machinery for the assimilation of sugars channelling them into lipid biosynthesis (“de novo” lipid accumulation) or diverting them into different carbohydrates which can promote their growth [47–59]. Starch and cellulose are the most abundant carbohydrates accumulated in microalgae and cyanobacteria cells [60, 61]. Starch accumulates in cytoplasm and chloroplasts. Cellulose accumulates mainly in cell walls representing a primary target when an additional carbon source is required in symbiotic associations of algae/cyanobacteria with other organisms [14]. In lichens, natural fungal-algal symbiotic associations, the fungal component can secrete hydrolytic enzymes to utilise microalgal cells walls during winter time when production of secreted carbohydrates from algae is limited because of suppressed photosynthesis [62]. Only a few cyanobacterial exopolysaccharides have been defined structurally, although some details of their composition are known [63, 64]. The sheaths of some of them contain cellulose-like homoglucan fibrils which are cross-linked by minor monosaccharides. The enzymatic cellulose degradation requires three types of enzymes: (1) endoglucanases, which break bonds in the crystalline structure of cellulose; (2) exoglucanases, which hydrolyse cellulose-forming free sugars or cellobiose; and (3) cellobiases, which hydrolyse intermediates generated by the action of these enzymes to free sugars [14]. Fungal cells can secrete a cocktail of hydrolytic enzymes containing cellulases along with hemicellulases, laccases and manganese peroxidase that in turn can convert cell wall polymers into reduced sugars that can then be utilized as a carbon source [65–71]. Production of microalgal cell wall-free protoplasts after co-cultivation of microalgal cells with *A. fumigatus* was recently shown [27, 46], and a correlation between enhanced biomass of the fungal-algal pellets and secretion of cell-wall degrading cellulases was also observed after co-cultivation of the different filamentous fungal strains with *C. vulgaris* [23–26, 29]. In our experiments *A. fumigatus*-SD100 pellets showed 1.4-fold increases in biomass compare to additive biomasses of the both components when grown as monoculture (Table 3). This was correlated with 1.3-fold increase in production of total lipids. This suggests that *A. fumigatus* may potentially utilize the cell wall carbohydrates or carbohydrates secreted from *Synechocystis* cells as a carbon source.

### Ex novo lipid production in *A. fumigatus*-*Synechocystis* associations

Some microorganisms can also use fatty acids and hydrophobic polymers, such as vegetable oils, industrial fats or fish oils as carbon sources. This process is called “ex novo lipid accumulation” [72–77]. Ex novo accumulation is often accompanied by the secretion of lipases that catalyse hydrolysis of hydrophobic polymers into FFA, which can then be transported into the cells using active transport mechanisms. If, however, the concentration of FFAs is high they can diffuse freely into the microbial cells [78, 79]. Absorbed FFAs can be converted and stored as TAGs and steryl esters incorporated into lipid bodies. Alternatively, FFAs can be directly used for cell growth and proliferation [80–83]. In the latter scenario FFAs can be converted into acyl–CoA esters by acyl–CoA synthetases followed by their degradation into smaller chain acyl–CoA ester and acetyl–CoA by the β–oxidation process, catalysed by various acyl–CoA oxidases providing the energy necessary for cell growth, maintenance and the production of intermediate metabolites [84]. Effects of exogenous FFAs on different microorganism’s growth were shown to vary extensively. Depending on their concentrations, composition and growth conditions (light/dark, aerobic/anaerobic), FFAs could inhibit or work as growth stimulating factors for different microorganisms [85–87].

The representatives of ‘FFA Secretion’ and ‘Green Recovery’ strains were observed to secrete substantial amounts of FFAs into the growth media. However, Nile Red staining and FFA extraction revealed no detectable FFAs in media after co-cultivation with *A. fumigatus*. There appeared to be a direct link between levels of secreted FFAs that accumulated in media before co-cultivation and total biomass and lipid yields in *A. fumigatus*-*Synechocystis* pellets. Total biomass and yields of lipids and intracellular FFAs that accumulated in *A. fumigatus*-*Synechocystis* pellets were higher than the additive yields of lipids and intracellular FFAs plus the extracellular FFAs that accumulated in cells and growth media prior to co-cultivation [83]. *A. fumigatus*-*Synechocystis* SD277 pellets showed a 2.7-fold increase in total biomass and 1.9-fold increase in accumulation of lipids and internal FFAs. It is unclear whether observed increases are only due to ex novo biosynthesis or other molecules including secreted carbohydrates or *Synechocystis* cell wall components be potentially used by fungal cells for growth and lipid production. This was indicated, however, that ex novo biosynthesis of lipid material cannot take place at the same time as the de novo process since FFAs can inhibit two key genes involved in de novo lipid accumulation: ATP-citrate lyase and fatty acid synthetases [88–90].

### Alternative carbon, nitrogen and phosphorus sources for growing *A. fumigatus*-*Synechocystis* associations

Application of alternative carbon sources from lignocellulosic waste for large scale fungal and algal biomass production has been explored extensively. Our recent studies have shown that *A. fumigatus* grown on 1 % TWS as the sole carbon source showed increasing growth rate compared to *A. fumigatus* grown on carbon-free media (Additional file 3: Figure S2, Additional file 4: Figure S3) [26–28]. *A. fumigatus*, however produced more biomass and higher lipid yields when grown on glucose. *A. fumigatus/*TWS cells showed efficient trapping of the microalgal and cyanobacterial strains within the first 48 h. There may be more than one reason why *A. fumigatus*/TWS-*Synechocystis* pellets showed lower values of total biomass and lipid production compare to *A. fumigatus*/GLU-*Synechocystis* pellets. These reasons include: (1) lower flocculation efficiency of *A. fumigatus*/TWS pellets; (2) lower lipid and FFA concentrations in *A. fumigatus*/TWS before and during co-cultivation and (3) potential negative effect of de novo on ex novo lipid production inhibited by reduced sugars and low molecular weight products of TWS’s digestion. The application of wheat straw biomass for fungal-assisted flocculation needs to be further optimised to improve their flocculation efficiency and reduce production of potentially toxic chemicals. This strategy offers a greater potential to improve the economics of fungal-cyanobacterial biotechnology for biofuel production.

For decades mono-cultured algal/cyanobacterial and fungal cells have been used extensively for recovery of N and P and microelements from a variety of wastewaters [91–99]. Efficient wastewater treatment by *A. fumigatus*/microalgal systems has previously been shown [26, 27, 41, 46]. Genetically modified Synechocystis sp. PCC 6803 expressing a novel lactate dehydrogenase gene involved in d-lactate biosynthesis a feedstock for food, pharmaceutical and plastic industries was growing on BG11 supplemented with alternative sources on N and P from wastewater from municipal sludge subjected to anaerobic digestion [100]. Obtained results showed that wastewater nutrients can enhance d-lactate synthesis by 40 % improving economics of this technology. In this present study it was shown that the co-cultivation of *A. fumigatus*-*Synechocystis* pellets produced a synergistic effect on absorption of ammonium and phosphates from diluted SWW. This synergistic effect of fungi and microalgae on nutrient removal from wastewater has great potential to be applied to pilot-scale wastewater-based wastewater system cultivated in continuous or semi-continuous mode.

## Conclusions

Metabolic engineering provides tools for the reprogramming of biochemical pathways and offers opportunities for generating organisms with tailored composition of essential molecules that can in turn be used directly as petrochemicals or can be converted into aviation and transportation fuels. The fungal-assisted harvesting of unicellular cyanobacteria *Synechocystis* PCC 6803 and its genetically modified derivatives described in this study may help resolving a number of challenges which large scale algal/cyanobacterial biotechnology is facing:

1. *Efficient harvesting of Synechocystis cells*. *A. fumigatus* cells can harvest up to 100 % of *Synechocystis* SD strains after 48 h of co-cultivation.
2. *Enhancement of total biomass, lipid production and optimization of fatty acids composition*. Fungal-*Synechocystis* pelletization showed synergetistic effects on total biomass and lipid production. The composition of FFAs in these associations can be tailored through co-cultivating of fungal cells with different cyanobacterial species.
3. *Carbon, nitrogen and phosphorus from waste stream biomass as an alternative, sustainable and renewable nutrient supply*. Use of alternative C, N and P sources from agricultural waste and wastewaters may potentially improve the economics of large scale biofuel production using cyanobacterial cells.
4. *Application of ex novo lipid biosynthesis for biofuel production*. Conventional, de novo production of lipids in fungal cells can be complemented with ex novo utilization of FFAs secreted from genetically modified *Synechocystis* strains.

## Methods

### Pelletization of *A. fumigatus* cells

Pelletization was achieved according to [28]. In brief, to achieve pelletization the spore solutions (1.5–2.0 × 10^7^ spores/L) were cultivated at 28 °C in the liquid fungal growth broth (FGB) containing 3 g/L peptone, 0.6 g/L KH_2_PO_4_, 0.001 g/L ZnSO_4_, 0.4 g/L K_2_HPO_4_, 0.005 g/L FeSO_4_, 0.5 g/L MnSO_4_, 0.5 g/L MgSO_4_. As a carbon source we used 20 g/L glucose (*A. fumigatus*/GLU) or 1 % acid pre-treatment of wheat straw (TWS, *A. fumigatus*/TWS) with a shaking speed of 150 rpm for 72 h.

### *Synechocystis* strains

All *Synechocystis* PCC 6803 strains were received from Professor Roy Curtiss III Arizona State University and have been described in [17, 35]. The strains were grown axenically at 25 °C in BG-11 medium [101] under continuous illumination (250 μmol photons m^−2^ s^−1^) and bubbled with 4 % CO_2_-enriched air. The details for growing SD culture under CO_2_-enriched and limited conditions were described in [17]. Growth rates were analysed by counting the cell numbers using a TC10™ Automated Cell Counter (BioRad) and by measuring OD_750_. For biomass analysis SD cultures were centrifuged at 6000*g* and then washed twice with sterile water and centrifuged again and dried at 65 °C. *Synechocystis* strains were grown in six flasks to a cell density 1.0 ± 0.8 × 10^9^ (see Additional file 5: Figure S4). From this stage, (*t* = 0), three flasks were mixed with *A. fumigatus* pellets for 24 h of co-cultivation (*A. fumigatus*-SD, t = 24 h). Another 3 flasks were continued growing for another 24 h (control, *t* = 24 h). For ‘Green Recovery’ experiments strains were grown till 1.0 ± 0.8 × 10^9^ in six flasks. Three days before mixing with fungal pellets bubbling with 4 % CO_2_ was stopped in all flasks and they were sealed with plastic wrap and rotated at 100 rpm under continuous illumination. Cell’s colour started turning from green to yellow. After 3 days, (*t* = 0), three flasks were mixed with *A. fumigatus* pellets for 24 h of co-cultivation (*A. fumigatus*-SD strains, *t* = 24 h) under CO_2_ depleted conditions. Another 3 flasks were continued growing for another 24 h also under CO_2_ depleted conditions (control, *t* = 24 h).

### Fungal-assisted flocculation of SD cells

Before mixing with *Synechocystis* strains *A. fumigatus* pellets were washed by sterile BG11 medium. *Synechocystis* cultures were precipitated, washed and resuspended till concentration of 1.0 ± 0.8 × 10^9^ cell/mL in BG11. The fungal-SD mixtures were shaken at 150 rpm for 48 h under constant light (200 µmol m^−2^ s^−1^) at 25 °C. Fungal and SD mono-cultures were also grown in BG11 media for 48 h as controls. All experiments were biologically replicated at least three times. Cell number, biomass and OD_750_ were measured at time 0, 24 and 48 h. *Synechocystis* cell samples were analysed 3 min after stopping rotation [46]. Flocculation efficiency (FE) was calculated based on changes in OD, cell numbers and in chlorophyll concentrations of uncaptured SD cells in the co-cultivation media at time 0 and 48 h later according to the following formula: ${\text{FE}}{\,}\% = \frac{A - B}{A} \times 100$, where *A* = OD, cell number at time 0; *B* = OD, cell number after 24 h after 48 h. EF_50_ is represented by amount of *A. fumigatus* (DW) required to flocculate 50 % of SD cells from 100 mL media containing 1.0 × 10^9^ cells after 48 h. The morphology of the fungal and algal cells and co-cultivation pellets was observed under bright field conditions using a Leica DM 2500 with the attached camera is a Leica DFC 310 FX.

### Nile Red staining

For Nile Red staining the algal cells, fungal cells and co-cultivated pellets were collected by centrifugation and re-suspended in 1 mL of 20 % DMSO containing 5 μL of Nile Red stock solution (0.10 mg/mL of Nile Red dissolved in acetone) and incubated at 50 °C with shaking at 150 rpm for 5 min. The stained pellets were then subjected to fluorescent microscopy analysis to observe the formation of lipid droplets in the co-cultivated cells using Leica DM 2500 with an attached camera Leica DFC 310 FX. Nile-Red filter: excitation at 543 nm, emission 555–650 nm.

### Lipid yield and fatty acid profile analysis

Extraction and analysis of lipid yield and FAME composition analysis of algal, fungal and fungal-algal pellets were performed using a method previously described [19, 102]. Secreted FFAs were separated from the culture medium by hexane according [17, 35]. In brief, 50 mL of culture was acidified by 1.0 mL 1 M H_3_PO_4_ containing 1.0 g NaCl, and extracted with 25 mL hexane. Intracellular FFAs and lipids, the cells were extracted by the Folch method [103]. The FFA samples were analyzed by GC [104].

### Zeta potential and cell size measurements

The zeta potential and cell size measurements of cells were obtained using a Nano-ZS/ZEN 3600. The zeta potentials were evaluated at a room temperature of 20 ± 1 °C. To analyse effect of co-cultivation of *Synechocystis* and *A. fumigatus* cells on their surface charges we mixed SD277 with *A. fumigatus* pellets and after 12 h 50 ml of co-cultivation media was collected, centrifuged at 10,000 rpm and filtered through 0.22 µM filter to remove *Synechocystis* and fungal cells and spores. To analyse zeta potential values of all components in this co-cultivation media, SD277 and *A. fumigatus* cells growing separately in monocultures were precipitated and resuspended in co-cultivation media. *A. fumigatus*-SD277 pellets were also analysed submerged in co-cultivation media. For each species, triplicate cultures were taken for measurements and for each data set, 10–20 readings were taken for each sample.

### Acid pre-treatment of wheat straw

One gram of fine powder (approximately 1 mm sin size) of dry wheat straw was mixed with 1 M sulphuric acid and autoclaved for 10 min at 121 °C, allowed to cool, filtered through Whatman No. 1 filter paper, then washed by 0.1 M sodium hydroxide followed by 10 times with sterile water. The powder was dried at 80 °C and added to the media to a final concentration of 1 %.

### Wastewater treatment

The anaerobically digested swine lagoon wastewater (ASW) was provided by Dr. J Hill, Termes Consulting Ltd, Melbourne. Swine wastewater was treated anaerobically. Wastewater samples were centrifuged to remove large particles, filtered through Whatman filter paper and autoclaved at 121 °C, allowed to cool to room temperature, and stored at 4 °C. The concentrations of NH_4_^+^-N and PO_4_^−3^-P in the ASW were 680.7 and 145.7 mg/L, respectively. The concentration of other inorganic nitrogen in the wastewater, such as NO_3_^−^-N was very low and not reported. Wastewater was diluted to 25 and 10 % with tap water. The fungal and fungal-SD pellets were harvested by filtration and 200 wet pellets were added to the 250 mL of wastewater (approximately, 1 g/L DW). The mixtures were shaken at 150 rpm for 48 h. Samples of growth media were analyzed for ammonia cations, nitrate and phosphate anions using an ion chromatography system Dionex ICS-1100 (Thermo Scientific, USA).

### Environmental scan electron microscopy analysis

For environmental scanning electron microscopy the samples were first dehydrated using gradual ethanol concentration starting from 10 to 100 %. Samples were soaked in ethanol concentration for 10 min before being transferred into higher concentration at room temperature. Dehydrated samples were subjected to the critical point dry facility for 10 min. The dried samples then allowed to gold coating for 60 s before the images were captured using FEI Quanta 200 SEM using environmental detector.

### Statistical analysis

All experiments in this study were conducted in triplicate. All data are expressed as mean ± standard deviation. The experimental data were subjected to the one-way analysis of variance (ANOVA) as implemented in the GraphPad InStat 3 statistics platform. Tukey simultaneous tests were conducted to determine the statistical differences between treatments. In order to ascertain that the observed variations in growth rates, efficiency of nutrients uptake and the yield of pyrolysis products were statistically significant, the probability (*P*) values were determined. A 95 % confidence level (*P* < 0.05) was applied for all analyses.

## Additional files

10.1186/s13068-015-0364-2 Artistic representation of two strategies designed for secretion of FFAs to growth media: ‘FFA Secretion’ and ‘Green Recovery’ from *Synechocystis* PCC 6803 cells. Text in red represents genetic modifications of SD100 strains.
10.1186/s13068-015-0364-2 Description of plasmids and genotypes of wild-type and genetically modified *Synechocystis* PCC 6803 strains. For more detailed description see [17, 35].
10.1186/s13068-015-0364-2 Pelletization of *A. fumigatus* grown in liquid media. (A) *A. fumigatus* pellets grown on FGB with no extra carbon source (left); with 1% TWS (middle) and with 20% glucose (right); (B) *A. fumigatus* pellets grown on FGB with 20% glucose (viewed from bottom); (C) *A. fumigatus* pellets grown on FGB with 20% glucose (left) and with 1% TWS (right). Scale=5mm.
10.1186/s13068-015-0364-2 Biomass production and lipid yields of *A. fumigatus* growing on different carbon sources. *A. fumigatus/*NEC*: A. fumigatus* grown on FGB with no extra carbon source; *A. fumigatus/*GLU*: A. fumigatus* grown on FGB with 20% glucose; *A. fumigatus/*TWS: *A. fumigatus* grown on FGB with 1% TWS. Significance levels: *) p<0.05; **) p<0.01.
10.1186/s13068-015-0364-2 Representation of flocculation experiments with ‘FFA Secretion’ (1) and ‘Green recovery’ (2) strains.
10.1186/s13068-015-0364-2 Evaluation of *Synechocystis* PCC 6803 growth rates in the media containing 5% and 20% of *A. fumigatus*/TWS media. (A) BG11 growth media containing 5% of *A. fumigatus*/TWS media; B) BG11 growth media containing 20% *A. fumigatus*/TWS media.
10.1186/s13068-015-0364-2 Environmental scanning electron microscopy of *A. fumigatus* and *A. fumigatus*-*Synechocystis* PCC 6803 associations. (A,B) *A. fumigatus* pellets growing in liquid media; (C) *A. fumigatus*-SD100 and (D) *A. fumigatus*-SD216 pellets.
10.1186/s13068-015-0364-2 Increase in biomass and lipid/Intracellular FFA yield in *A. fumigatus-Synechocystis* pellets after flocculation. (A) Increase in lipids in *A. fumigatus/*GLU-SD pellets compare to the additive yields of lipid/intracellular FFA and extracellular FFAs extracted from mono-cultured *A. fumigatus* and SD strains before co-cultivation. (B) Increase in lipids in *A. fumigatus/*TWS-SD pellets compare to the additive yields of lipid/intracellular FFAs and extracellular FFAs extracted from mono-cultured *A. fumigatus* and SD strains before co-cultivation. Significance levels: *) p<0.05; **) p<0.01.
10.1186/s13068-015-0364-2 The samples of *A. fumigatus*-SD100, *A. fumigatus*-SD256 and *A. fumigatus*-SD257 pellets. 1) *A. fumigatus*-SD100 (left), *A. fumigatus*-SD256 after CO_2_ limitation (right); 2) *A. fumigatus*-SD100 (left), *A. fumigatus*-SD257 after CO_2_ limitation (right).
10.1186/s13068-015-0364-2 Application of *A. fumigatus*-SD100 pellets for 25% swine wastewater treatment. (1) 25% swine wastewater at t=0 and 48 h after treatment with SD100 (2); *A. fumigatus* (3) and *A. fumigatus*-SD (4). Tap water as control (5).

## Abbreviations

*A. fumigatus*: *Aspergillus fumigatus*
ACP: acyl–acyl carrier protein
*C. camphorum*: *Cinnamomum camphorum*
*C. hookeriana*: *Cuphea hookeriana*
C: carbon
DAG: diacylglycerols
FE_50_: half maximal flocculation efficiencies
FFA: free fatty acids
GLU: glucose
N: nitrogen
NEC: no extra carbon
P: phosphate
SWW: swine wastewater
TAG: triacylglycerols
TE: thioesterases
TWS: treated wheat straw
*U. californica*: *Umbellularia californica*
