# Supplementary material for: Lipid production in association of filamentous fungi with genetically modified cyanobacterial cells
Source: Biotechnol Biofuels. 2015 Nov 5;8:179. doi: 10.1186/s13068-015-0364-2 (PMC4635583; doi:10.1186/s13068-015-0364-2)
Supplement: Supplementary file 3 [file 13068_2015_364_MOESM2_ESM.docx]

Table S1: Description of plasmids and genotypes of generated *Synechocystis* PCC6803 strains.#

Constructs	Genotype	Description of vectors
SD100		*Synechocystis* sp. PCC 6803 wild type
	'FFA Secretion'	
SD216	*Δslr1609::PpsbA2'tesA*	*E. coli* 'tesA gene is driven by PpsbA2 promoter. Deletion of an *acyl-ACP* synthesis gene, (*slr1609*) prevents FFA's channelling into a number of competitive pathways.
SD232	*Δslr1609::PpsbA2'tesA Δ(slr1993-slr1994)::PcpcaccBC PrbcaccDA Δsll1951::PpsbA2 Ucfatb1 Prbc Ch fatB2*	Overexpression of *E. coli* 'tesA gene and plant ACP thioesterases from *Umbellularia californica* (12:0) and *Cuphea hookeriana* (C8:0 and C10:0). Deletion of surface structure gene (*sll1951*). Deletion of an acyl-ACP synthesis gene, (slr1609).
SD277	*Δslr1609::PpsbA2'tesA Δ(slr1993-slr1994)::PcpaccBC Prbc accDA Δsll1951::PpsbA2 Uc fatB1 Prbc Ch fatB2Δ(slr2001-slr2002)::PpsbA2 ChfatB2Δslr1710::PpsbA2 Cc fatB1 Δslr2132::PtrctesA137*	Overexpression of codon-optimized tesA137 gene and plant ACP thioesterases from *Cinnamomum camphorum* (14:0), *Umbellularia californica* (12:0) and *Cuphea hookeriana* (C8:0 and C10:0). Deletion of surface structure gene (*sll1951*). Deletion of surface structure gene (sll1951). Deletion of an acyl-ACP synthesis gene, (slr1609).
	'Grean Recovery'	
SD256	*Pcmp::fol*	Expression of lipolytic enzyme from *Fusarium oxysporum* under control of CO2-limitation-inducible promoter.
SD257	*Pcmp::shl*	Expression of lipolytic enzyme from *Staphylococcus hyicus* under control of CO2-limitation-inducible promoter
SD277	*Δslr1609::PpsbA2'tesA Δ(slr1993-slr1994)::Pcpc accBC Prbc accDA Δsll1951::'PpsbA2 Uc fatB1 Prbc Ch fatB2 Pcmp::fol RBS shl Pabt::gpl RBS 13 19 15*	Overexpression of *E. coli* 'tesA gene; plant acyl–acyl carrier protein (ACP) thioesterases from *Umbellularia californica* (12:0) and *Cuphea hookeriana* (C8:0 and C10:0). Deletion of surface structure gene (*sll1951*). Deletion of an acyl-ACP synthesis gene, (slr1609). Expression of fused lipolytic enzymes from *Staphylococcus hyicus* and *Fusarium oxysporum* under control of CO2-limitation-inducible promoter

#For more detailed description see [16, 34]

Additional file 2: Table S1
