# Supplementary material for: Lipid production in association of filamentous fungi with genetically modified cyanobacterial cells
Source: Biotechnol Biofuels. 2015 Nov 5;8:179. doi: 10.1186/s13068-015-0364-2 (PMC4635583; doi:10.1186/s13068-015-0364-2)
Supplement: Supplementary file 10 [file 13068_2015_364_MOESM9_ESM.docx]

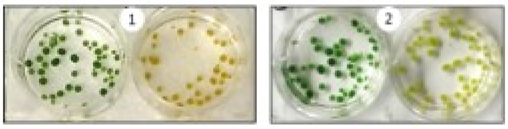

Additional file 9. Figure S8
